# The mortality burden in patients with hip fractures and dementia

**DOI:** 10.1007/s00068-021-01612-4

**Published:** 2021-02-27

**Authors:** Ioannis Ioannidis, Ahmad Mohammad Ismail, Maximilian Peter Forssten, Rebecka Ahl, Yang Cao, Tomas Borg, Shahin Mohseni

**Affiliations:** 1 Department of Orthopedic Surgery, Orebro University Hospital, 701 85 Orebro, Sweden; 2 School of Medical Sciences, Orebro University, 702 81 Orebro, Sweden; 3 Division of Trauma & Emergency Surgery, Department of Surgery, Karolinska University Hospital, 171 76 Stockholm, Sweden; 4 Clinical Epidemiology and Biostatistics, School of Medical Sciences, Orebro University, 701 82 Orebro, Sweden; 5 Division of Trauma and Emergency Surgery, Department of Surgery, Orebro University Hospital, 701 85 Orebro, Sweden

**Keywords:** Hip fracture, Dementia, Postoperative mortality, Cause-specific mortality

## Abstract

**Purpose:**

Dementia is strongly associated with postoperative death in patients subjected to hip fracture surgery. Nevertheless, there is a distinct lack of research investigating the cause of postoperative mortality in patients with dementia. This study aims to investigate the distribution and the risk of cause-specific postoperative mortality in patients with dementia compared to the general hip fracture population.

**Methods:**

All adults who underwent emergency hip fracture surgery in Sweden between 1/1/2008 and 31/12/2017 were considered for inclusion. Pathological, conservatively managed fractures, and reoperations were excluded. The database was retrieved by cross-referencing the Swedish National Quality Registry for Hip Fracture patients with the Swedish National Board of Health and Welfare quality registers. A Poisson regression model was used to determine the association between dementia and all-cause as well as cause-specific 30-day postoperative mortality.

**Results:**

134,915 cases met the inclusion criteria, of which 20% had dementia at the time of surgery. The adjusted risk of all-cause 30-day postoperative mortality was 67% higher in patients with dementia after hip fracture surgery compared to patients without dementia [adj. IRR (95% CI): 1.67 (1.60–1.75), *p* < 0.001]. The risk of cause-specific mortality was also higher in patients with dementia, with up to a sevenfold increase in the risk cerebrovascular mortality [adj. IRR (95% CI): 7.43 (4.99–11.07), *p* < 0.001].

**Conclusions:**

Hip fracture patients with dementia have a higher risk of death in the first 30 days postoperatively, with a substantially higher risk of mortality due to cardiovascular, respiratory, and cerebrovascular events, compared to patients without dementia.

## Background

The association between dementia and an increased risk of mortality after hip fracture operations is strongly backed by scientific evidence in the field of orthopedic surgery [1–4]. The volume of hip fractures is projected to continue to grow globally due to an aging population [5]. Currently, the cumulative lifetime risk of suffering a hip fracture after the age of 50 is 24% in women and 11% in men in Sweden [6]. In addition to other medical conditions which are associated with worse postoperative outcomes, up to 23% of hip fracture patients suffer from dementia [7, 8]. By 2050, the prevalence of dementia is projected to have doubled [9]. These numbers are consistent with those predicted worldwide [10].

There is a paucity in research that studies the cause of death in patients with dementia after hip fracture surgery along with how it relates to the general hip fracture population without dementia. To pinpoint interventions that may be used for reducing mortality in hip fracture patients with dementia further research into cause-specific mortality is warranted. Rather than merely focusing on all-cause mortality in this patient population, this study aims to investigate the distribution and the risk of cause-specific mortality in hip fracture patients with dementia compared to the general hip fracture population.

## Methods

After obtaining approval from the Swedish Ethical Review Authority (reference 2020-04161), all adult cases of hip fracture surgery, between January 1, 2008 and December 31, 2017, were retrieved from the prospectively collected Swedish National Quality Registry for Hip Fracture Patients, Rikshöft [11]. Patients whose hip fracture was managed non-operatively, pathological fractures, and reoperations were excluded from the current study. The following data were retrieved from Rikshöft: date of hospital admission, age, sex, fracture type, American Society of Anesthesiologist (ASA) classification, surgical method, date of surgery, and hospital discharge date. The data from Rikshöft were cross-referenced with the Swedish National Board of Health and Welfare Patient and Cause of Death registers by matching patients’ unique social security numbers, which allowed for the retrieval of data pertaining to time of death and comorbidities. All diagnoses registered for each social security number in the patient register, including dementia, were included as comorbidities. The comorbidity data were used to calculate the Charlson Comorbidity Index (CCI) [12]. The principles of the Declaration of Helsinki and STROBE guidelines were adhered to while conducting this study [13].

### Statistical analysis

The cases were divided into two cohorts: patients with and without dementia. Demographics and clinical characteristics were compared between the cohorts; categorical variables are reported as percentages and continuous variables are reported as a mean and standard deviation (SD) or median and interquartile range (IQR). The statistical significance of differences between categorical variables was measured using Pearson’s Chi-squared test. For continuous variables, the Student’s *t* test was used for normally distributed data, otherwise the Mann–Whitney *U* test was applied. The primary outcome of interest was 30-day postoperative mortality.

A Poisson regression model was used to determine the association between dementia and 30-day all-cause postoperative mortality. The existence of hypertension, arrhythmias, peptic ulcer disease, and hemiplegia are reported in the cohort demographics but not adjusted for in the multivariate regression analyses. Hypertension and arrhythmia were not included since they are risk factors for other diagnoses, which were already adjusted for in the model, such as myocardial infarctions, congestive heart failure, and cerebrovascular events. Peptic ulcer disease was excluded as a result of its negligible association with mortality [14]. Adjusting for hemiplegia would risk overfitting the model since hemiplegia is often a result of a cerebrovascular event. The analysis was repeated to calculate the incidence rate ratio (IRR) for 30-day *cause-specific* mortality for hip fracture patients with dementia compared to those without dementia. All analyses were performed while adjusting for age, sex, ASA classification, fracture type, type of surgery, and relevant comorbidities (prior myocardial infarctions, prior cerebrovascular events, peripheral vascular disease, chronic obstructive pulmonary disease, congestive heart failure, connective tissue diseases, diabetes mellitus, liver disease, chronic kidney disease, as well as local tumor and metastatic carcinoma). Multivariate imputation by chained equations was implemented to compensate for missing data in the covariates used in the regression model; logistic regression was used for binary variables, Bayesian polytomous regression for nominal variables, and a proportional odds model for ordinal variables.

Results are reported as IRRs with 95% confidence intervals (CI). Statistical significance was defined as a two-sided *p* value of less than 0.05. Analyses were performed using the statistical programming language R (R Foundation for Statistical Computing, Vienna, Austria) [15].

## Results

A total of 134,915 cases met the study inclusion criteria. Patients with dementia tended to be older [mean (SD): 85 (11) vs 81 (7) years, *p* < 0.001] and females were more prevalent in both cohorts (dementia: 69.2%, no dementia: 67.9%, *p* < 0.001). Patients with dementia were less fit for surgery (ASA ≥ 3: 71.2% vs 54.0%, *p* < 0.001) and had more comorbidities (CCI ≥ 7: 28.5% vs 16.0%, *p* < 0.001) (Table 1). Hemiarthroplasty was undertaken to a larger extent in patients with dementia (31.4% vs 24.2%, *p* < 0.001) while total hip replacement was more common in patients without dementia (8.7% vs 2.0%, *p* < 0.001). There were no clinically significant differences in the distribution of fracture types (Table 1). Depicted in Table 2 are the prevalence of specific comorbidities within each cohort. Arrhythmia, congestive heart failure, cerebrovascular events, and peptic ulcer disease were more prevalent in patients with dementia, while peripheral vascular disease, chronic obstructive pulmonary disease, connective tissue disease, liver disease, diabetes mellitus, hemiplegia, chronic kidney disease, local tumor, and metastatic carcinoma were more common in patients without dementia (Table 2).

**Table 1:** Patient demographics and clinical characteristics in hip fracture cases undergoing surgery with and without dementia

	No dementia *N* = 107,611	Dementia *N* = 27,304	*p* value
Age in years, mean [SD]	81 [11]	85 [7]	< 0.001
Sex, *n* (%)			< 0.001
Female	73,032 (67.9)	18,881 (69.2)	
Male	34,566 (32.1)	8,422 (30.8)	
Missing	13 (0.0)	1 (0.0)	
ASA classification, *n* (%)			< 0.001
1	6,359 (5.9)	297 (1.1)	
2	41,227 (38.3)	7,037 (25.8)	
3	50,120 (46.6)	16,737 (61.3)	
4	7,866 (7.3)	2,668 (9.8)	
5	95 (0.1)	40 (0.1)	
Missing	1,944 (1.8)	525 (1.9)	
Charlson comorbidity index, *n* (%)			< 0.001
≤ 4	57,030 (53.0)	2,581 (9.5)	
5–6	33,313 (31.0)	16,934 (62.0)	
≥ 7	17,268 (16.0)	7,789 (28.5)	
Fracture type, *n* (%)			< 0.001
Non-displaced cervical (Garden 1–2)	14,205 (13.2)	3,663 (13.4)	
Displaced cervical (Garden 3–4)	39,789 (37.0)	10,383 (38.0)	
Basicervical	3,541 (3.3)	939 (3.4)	
Pertrochanteric (two fragments)	21,779 (20.2)	5,080 (18.6)	
Pertrochanteric (multiple fragments)	19,206 (17.8)	5,287 (19.4)	
Subtrochanteric	9,044 (8.4)	1,944 (7.1)	
Missing	47 (0.0)	8 (0.0)	
Type of surgery, *n* (%)			< 0.001
Pins or screws	18,585 (17.3)	4,873 (17.8)	
Screws or pins with sideplate	28,260 (26.3)	6,642 (24.3)	
Intramedullary rod	25,353 (23.6)	6,639 (24.3)	
Hemiarthroplasty	26,015 (24.2)	8,581 (31.4)	
Total hip replacement	9,332 (8.7)	557 (2.0)	
Missing	66 (0.1)	12 (0.0)	

*SD* standard deviation, *ASA* American Society of Anesthesiologists

**Table 2:** Preoperative comorbidities in hip fracture cases undergoing surgery with and without dementia

	No dementia *N* = 107,611	Dementia *N* = 27,304	*p* value
Hypertension, *n* (%)	41,294 (38.4)	10,462 (38.3)	0.87
Arrhythmia, *n* (%)	19,755 (18.4)	5,243 (19.2)	< 0.001
Myocardial infarction, *n* (%)	6,452 (6.0)	1,611 (5.9)	0.56
Congestive heart failure, *n* (%)	16,525 (15.4)	4,572 (16.7)	< 0.001
Peripheral vascular disease, *n* (%)	4,943 (4.6)	947 (3.5)	< 0.001
Cerebrovascular event, *n* (%)	17,477 (16.2)	5,905 (21.6)	< 0.001
Chronic obstructive pulmonary disease, *n* (%)	12,841 (11.9)	2,736 (10.0)	< 0.001
Connective tissue disease, *n* (%)	5,412 (5.0)	1,075 (3.9)	< 0.001
Peptic ulcer disease, *n* (%)	3,397 (3.2)	931 (3.4)	0.036
Liver disease, *n* (%)	1,185 (1.1)	185 (0.7)	< 0.001
Diabetes mellitus, *n* (%)	15,987 (14.9)	3,869 (14.2)	0.004
Hemiplegia, *n* (%)	2,441 (2.3)	470 (1.7)	< 0.001
Chronic kidney disease, *n* (%)	5,621 (5.2)	1,324 (4.8)	0.013
Local tumor, *n* (%)	11,893 (11.1)	2,667 (9.8)	< 0.001
Metastatic carcinoma, *n* (%)	2,595 (2.4)	367 (1.3)	< 0.001

Crude 30-day all-cause postoperative mortality was twice as high in patients with dementia compared to those without dementia (12.8% vs 6.2%, *p* < 0.001). All specific causes of death were also more prevalent in patients with dementia (Table 3). In the Poisson regression analysis, the incidence of 30-day mortality was 67% higher in hip fracture patients with dementia [adj. IRR (95% CI): 1.67 (1.60–1.75), *p* < 0.001] (Table 4). Patients with dementia had a 42% [adj. IRR (95% CI): 1.42 (1.32–1.54), *p* < 0.001] and a 76% [adj. IRR (95% CI): 1.76 (1.57–1.99), *p* < 0.001] increased risk of death caused by postoperative cardiovascular and respiratory events, respectively. The risk of death due to sepsis was twice as high in patients with dementia [adj. IRR (95% CI): 2.17 (1.47–3.22), *p* < 0.001]. Patients with dementia also had a sevenfold increase in the risk of death resulting from cerebrovascular events [adj. IRR (95% CI): 7.43 (4.99–11.07), *p* < 0.001] (Table 4).

**Table 3:** Crude 30-day postoperative mortality in hip fracture cases undergoing surgery with and without dementia

	No dementia *N* = 107,611	Dementia *N* = 27,304	*p* value
All-cause mortality			
30-day mortality, *n* (%)	6,640 (6.2)	3,490 (12.8)	< 0.001
Cause-specific mortality			
Cardiovascular, *n* (%)	2,895 (2.7)	1,287 (4.7)	< 0.001
Respiratory, *n* (%)	1,083 (1.0)	619 (2.3)	< 0.001
Cerebrovascular, *n* (%)	58 (0.1)	108 (0.4)	< 0.001
Sepsis, *n* (%)	118 (0.1)	74 (0.3)	< 0.001
Multi-organ failure, *n* (%)	2,413 (2.2)	1,176 (4.3)	< 0.001
Unknown, *n* (%)	73 (0.1)	226 (0.8)	< 0.001

**Table 4:** Incidence rate ratio (IRR) for postoperative mortality after hip fracture surgery

Variable	30-day mortality IRR (95% CI)	*p* value
All-cause mortality		
No dementia	Ref	
Dementia	1.67 (1.60–1.75)	< 0.001
Cause-specific mortality for patients with dementia*		
Cardiovascular	1.42 (1.32–1.54)	< 0.001
Respiratory	1.76 (1.57–1.99)	< 0.001
Cerebrovascular**	7.43 (4.99–11.07)	< 0.001
Sepsis	2.17 (1.47–3.22)	< 0.001
Multi-organ failure	1.66 (1.53–1.79)	< 0.001
Unknown***	11.2 (8.06–15.58)	< 0.001

Poisson regression model with robust standard errors. Multiple imputation with chained equations was used to manage missing values. The model is adjusted for age, sex, American Society of Anesthesiologists (ASA) classification, fracture type, type of surgery, prior myocardial infarctions, prior cerebrovascular events, peripheral vascular disease, chronic obstructive pulmonary disease, congestive heart failure, connective tissue diseases, diabetes mellitus, liver disease, chronic kidney disease, as well as local tumor and metastatic carcinoma

^*^Reference group is patients without dementia

^**^Not adjusted for ASA classification and liver disease due to their inclusion resulting in a matrix that is computationally singular

^***^Not adjusted for ASA classification due to its inclusion resulting in a matrix that is computationally singular

## Discussion

To the authors’ best knowledge, this is the first study investigating the risk of all-cause and cause-specific mortality in hip fracture patients with dementia compared to the general hip fracture population. In this large nationwide cohort study, patients with dementia had twice the risk of postoperative all-cause mortality compared to patients without dementia. After adjusting for differences between the cohorts, there was a substantially increased risk of death due to cardiovascular, pulmonary, cerebrovascular, and septic events in patients with dementia.

The relationship between dementia and postoperative mortality has long been known by orthopedic surgeons [1–4]. In a meta-analysis by Rao et al. from 2016, comparing hospital outcomes in all patients with and without dementia, the authors found that the relative risk of all-cause mortality was 70% higher in patients with dementia. This corresponded to an overall mortality rate of 15.3% in dementia patients compared to 8.7% in non-dementia cases. Patients with dementia also had higher readmission rates and longer lengths of stay in the hospital. However, they also underwent fewer life-saving interventions and procedures [16]. Similar results were found in our study where the relative risk of all-cause mortality was increased by 67% in patients with dementia. The crude mortality rate was 12.8% in patients with dementia compared to 6.2% in patients without dementia. To be able to pinpoint which interventions aimed at reducing mortality are worth investigating, research into the specific causes of mortality is required. Previous studies have primarily been concerned with the distribution of causes of mortality in patients who died with dementia, as opposed to the risk for such events compared to patients without dementia [17, 18].

Dementia has often been referred to as the cause of mortality in previous, often orthopedic, studies [18–22]. The current study demonstrates that this line of reasoning is misleading. Patients with dementia have a higher risk of postoperative death, but they still die from the failure of a specific organ or multiple organ systems. Labeling the cause of death as dementia obscures this fact and lulls physicians into a false sense of security by promoting the reasoning ‘there was nothing more we could have done’. Dementia is a risk factor that predicts mortality, not the end cause [2–4].

Hip fracture patients with dementia should be considered a distinct patient population in orthopedic surgery in need of particular care [2, 16]. In light of this, the use of risk stratification tools in the clinical setting may be of interest. Several tools have been investigated in the context of the general hip fracture population, such as the Nottingham Hip Fracture Score, CCI, and Physiological and Operative Severity Score for the enUmeration of Mortality and Morbidity (POSSUM) [12, 23–25]. Risk stratification may aid in attaining better resource allocation, patient prioritization, preoperative optimization, and monitoring. More significantly, it can assist orthopedic surgeons and their multidisciplinary teams in identifying patients who might gain from specialized postoperative care.

Old age, frailty, polypharmacy, comorbidities, and dementia are well-known risk factors for delirium in hospital patients, particularly postoperative delirium [26–29]. All these factors are common in hip fracture patients [7, 8, 19, 30–34], and even more so in those with dementia, resulting in delirium being a familiar complication on orthopedic wards. Postoperative delirium episodes have been strongly linked to an increased mortality risk, especially in the emergency setting [28]. As it stands, multiple guidelines have been proposed recently to reduce the risk of delirium in geriatric patients, which may warrant further examination and further adjustment for patients with dementia [35–37]. Reducing the risk of delirium could potentially decrease the risk of many of the specific causes of death after hip fracture surgery, particularly in the more vulnerable population of patients with dementia.

Due to the retrospective nature of the current study, the authors cannot make any certain claims about the increased risk of respiratory death detected. The high proportion of respiratory deaths has previously been noted in the general dementia population which is consistent with the current findings [17, 18]. This is an important finding since postoperative measures to decrease the risk of respiratory complications, which have a higher incidence postoperatively, could be emphasized more in this patient population. Further investigations are required to determine which perioperative interventions are needed to reduce each specific postoperative mortality risk in hip fracture patients with dementia.

This study is based on 10 consecutive years of data from the Swedish National Quality Registry for Hip Fracture Patients, which is known for having a high case coverage between 80 and 90% [38]. As a result of the universal healthcare system in Sweden, patient management is also relatively uniform across all orthopedic departments. Furthermore, the nature of this system removes many of the socioeconomic barriers that might result in some patients going undiagnosed and undertreated for serious health conditions. However, several limitations to the current study are worth mentioning. Due to the retrospective nature of the study, surgical experience, postoperative pain, and functional outcome could not be included in the analysis. In addition, while dementia has been treated as a single monolithic disease in the current study, the reality is that dementia is the result of several different conditions with varying pathophysiology and mortality rates [17, 18]. The authors recognize that it is possible that a distinction between these different conditions could result in different mortality rates.

## Conclusion

This study demonstrates that hip fracture patients with dementia have a higher risk of death in the first 30 days postoperatively, with a substantially higher risk of cardiovascular, respiratory, and cerebrovascular mortality specifically. These findings could be used as the basis for better risk assessments in this patient population, and aid improved perioperative monitoring and care optimization, to achieve better overall outcomes after hip fracture surgery.

## Data Availability

All codes are available upon reasonable request.
